# Family specific genetic predisposition to breast cancer: results from Tunisian whole exome sequenced breast cancer cases

**DOI:** 10.1186/s12967-018-1504-9

**Published:** 2018-06-07

**Authors:** Yosr Hamdi, Maroua Boujemaa, Mariem Ben Rekaya, Cherif Ben Hamda, Najah Mighri, Houda El Benna, Nesrine Mejri, Soumaya Labidi, Nouha Daoud, Chokri Naouali, Olfa Messaoud, Mariem Chargui, Kais Ghedira, Mohamed Samir Boubaker, Ridha Mrad, Hamouda Boussen, Sonia Abdelhak, the PEC Consortium

**Affiliations:** 1 Laboratory of Biomedical Genomics and Oncogenetics, LR16IPT05, Institut Pasteur de Tunis, University of Tunis El Manar, 13, Place Pasteur-BP 74, 1002 Tunis, Tunisia; 2 Laboratory of Bioinformatics, Biomathematics and Biostatistics, LR16IPT09, Institut Pasteur de Tunis, University of Tunis El Manar, Tunis, Tunisia; 3 Faculty of Sciences of Bizerte, Carthage University, Tunis, Tunisia; 4 Department of Medical Oncology, Abderrahmane Mami Hospital, Ariana, Tunisia; 5 Department of Human Genetics, Charles Nicolle Hospital, Tunis, Tunisia

**Keywords:** Breast cancer, Exome sequencing, Family specific predisposition, Non BRCA Tunisian families

## Abstract

**Background:**

A family history of breast cancer has long been thought to indicate the presence of inherited genetic events that predispose to this disease. In North Africa, many specific epidemio-genetic characteristics have been observed in breast cancer families when compared to Western populations. Despite these specificities, the majority of breast cancer genetics studies performed in North Africa remain restricted to the investigation of the *BRCA1* and *BRCA2* genes. Thus, comprehensive data at a whole exome or whole genome level from local patients are lacking.

**Methods:**

A whole exome sequencing (WES) of seven breast cancer Tunisian families have been performed using a family-based approach. We focused our analysis on BC-TN-F001 family that included two affected members that have been sequenced using WES. Relevant variants identified in BC-TN-F001 have been confirmed using Sanger sequencing. Then, we conducted an integrative analysis by combining our results with those from other WES studies in order to figure out the genetic transmission model of the newly identified genes. Biological network construction and protein–protein interactions analyses have been performed to decipher the molecular mechanisms likely accounting for the role of these genes in breast cancer risk.

**Results:**

Sequencing, filtering strategies, and validation analysis have been achieved. For BC-TN-F001, no deleterious mutations have been identified on known breast cancer genes. However, 373 heterozygous, exonic and rare variants have been identified on other candidate genes. After applying several filters, 12 relevant high-risk variants have been selected. Our results showed that these variants seem to be inherited in a family specific model. This hypothesis has been confirmed following a thorough analysis of the reported WES studies. Enriched biological process and protein–protein interaction networks resulted in the identification of four novel breast cancer candidate genes namely *MMS19, DNAH3*, *POLK* and *KATB6.*

**Conclusions:**

In this first WES application on Tunisian breast cancer patients, we highlighted the impact of next generation sequencing technologies in the identification of novel breast cancer candidate genes which may bring new insights into the biological mechanisms of breast carcinogenesis. Our findings showed that the breast cancer predisposition in non-*BRCA* families may be ethnic and/or family specific.

**Electronic supplementary material:**

The online version of this article (10.1186/s12967-018-1504-9) contains supplementary material, which is available to authorized users.

## Background

A range of genetic and non-genetic risk factors contribute to the development of breast cancer [1]. So far, several genetic variants of high, moderate and low penetrance have been identified as impacting on breast cancer risk using familial linkage, DNA resequencing and genome wide association analysis, respectively [2]. The identification of additional breast cancer associated genes is crucial to explain the missing breast cancer heritability. Recent studies showed that breast cancer susceptibility may be explained by a polygenic risk model of inheritance in which a large number of common SNPs contribute multiplicatively towards risk [3]. With the introduction of next generation sequencing (NGS) technologies [4, 5] many studies suggested that a large rate of the remaining breast cancer heritability can be attributed to new rare risk alleles that segregate in an autosomal-dominant pattern of inheritance.

To date, two different whole exome sequencing study designs are used: case/control association studies and the family-based approach. The case/control design is considered as the major promising tool to detect significant associations between genetic variations and breast cancer disease [6]. However, due to the extreme rarity of certain variants, this approach requires large-size cohorts to confirm the association between these variants and breast cancer risk. The second WES design is the family-based approach [7] where breast cancer family members are exome-sequenced and the shared variants between affected individuals presumably include the familial breast cancer risk allele. Thus, focusing on the family segregation of relevant variants is expected to better detect novel susceptibility variants than the screening of pooled unrelated cases and controls.

Several WES studies have been performed on hereditary breast cancer [7, 8]. Almost, 108 breast cancer families have been whole exome sequenced using the family-based approach and reported many relevant variants present in related affected individuals and absent in unaffected ones. So far, five new genes have been identified by WES as associated with breast cancer risk, among them four genes identified using the family-based approach, namely: *XRCC2* [9], *MAPKAP1* [10], *FANCM* [11] and *RINT1* [12] while only one gene, *REQCL*, was identified using the case/control approach [13]. Mutations on known breast cancer susceptibility genes were reported in only four families [10–14].

In Tunisia, breast cancer is the most common and the most deadly form of cancer among females [15]. Several epidemiological, genetic and clinical breast cancer characteristics have been observed to be unique to Tunisian and North African population. Indeed, breast cancer shows a lower incidence rate but a younger age of disease onset, when compared to Western populations, with a relative high frequency of the aggressive breast cancer forms such as inflammatory and triple negative breast cancers [16]. Thus, a genetic predisposition specific to this ethnic group is plausible, [8, 17, 18]. Moreover, it is possible that breast cancer risk variants are so rare that they are “family specific” meaning that a genetic predisposition can be detected within a disease-prone family, but not necessarily shared with other genetically unrelated families with the same disease [19–21].

So far, genetic studies performed on Tunisian breast cancer patients mostly focused on the *BRCA* genes using the traditional Sanger technique. Therefore, the use of next generation sequencing technologies in the genetic investigation of these under-exploited populations may help identifying novel breast cancer risk allele and explain the remaining unresolved breast cancer genetic heritability.

In the present study, we performed a whole exome sequencing of seven BRCAx breast cancer Tunisian families with strong family history in order to identify genetic variations that may be associated with breast cancer risk. Using the family-based approach, we focused our analysis on a non BRCA family by sequencing two out of three affected sisters. After comparing our results to those identified in previous WES studies and by performing biological network analysis, we identified a set of novel breast cancer candidate genes that seems to be inherited in a family specific manner.

## Methods

### Patients

Seven Tunisian breast cancer families were selected for WES based on the following criteria: (1) Presence of at least three related first or second-degree breast cancer cases; (2) Breast cancer in young patients aged less than 35 years, (3) Presence of at least two cases of breast or ovarian cancer, regardless of age, and at least one case of pancreatic cancer or prostate cancer in a related first or second degree patient. Blood samples have been collected from the affected family members and have been sampled in the Medical oncology department, Abderrahman Mami Hospital, Ariana, Tunisia. Written informed consents were obtained from all participants. Ethical approval according to the Declaration of Helsinki Principles was obtained from the biomedical ethics committee of Institut Pasteur de Tunis (2017/16/E/Hôpital a-m/V1).

Two out of three affected sisters from BC-TN-F001 have been whole exome sequenced. The proband was diagnosed with a primary breast cancer at age 43 and contralateral invasive ductal breast carcinoma at age 48. The second family member involved in this study was diagnosed with an invasive breast cancer at age 56. Phenotypic characteristics of the affected family members are described in Table 1.

**Table 1:** Epidemiological and clinical data of affected family members

Family Member	Diagnosis age	Histological subtype	SBR grade	Tumor size (mm)	Hormone receptors status	HER2 status	Disease evolution	Medical history
BC-TN-F001-1	43	Invasive ductal carcinoma	II	22	ER +/PR+	ND	CBC within 5 years, grade III triple negative carcinoma	3 miscarriages
BC-TN-F001-2	56	Invasive ductal carcinoma	ND	ND	ER +/PR+	ND	In remission	No medical history
BC-TN-F001-3	47	Bifocal invasive ductal carcinoma	I	7	ER +/PR+	HER2−	In remission	Primary infertility (IVF)

*CBC* contralateral breast cancer; *ER* estrogen receptor; *PR* progesterone receptor; *ND* not determined; *IVF* in vitro fertilization

### Whole exome sequencing and data analysis

For each participant, total genomic DNA was isolated from peripheral blood using the salting out method or the DNeasy blood Kit from Qiagen according to the manufacturer’s instructions. DNA purity and concentration were measured using a NanoDrop™ spectrophotometer.

Samples were prepared according to Agilent’s SureSelect Protocol version 1.2 and enrichment was carried out according to Agilent SureSelect protocols. Enriched samples were sequenced on the Illumina HiSeq 2000 platform using TruSeq v3 chemistry with paired-end (2 × 100pb).

Exome DNA sequences were mapped to their location in the build of the human genome (hg19/b37) using the Burrows–Wheeler Aligner (BWA) package. The subsequent SAM files were converted to BAM files using Samtools. Duplicate reads were removed using Picard. GATK was then used to recalibrate the base quality scores as well as for SNP and short INDEL calling. Annotation and prioritization of potential disease-causing variants were performed using VarAFT (Variant Annotation and Filtering Tool) (http://varaft.eu). To annotate variants, VarAFT uses ANNOVAR, a command line tool. INDELs and SNPs annotated were filtered according to several criteria: (1) considering breast cancer as autosomal dominant disease and removing variants that were found in a homozygous state, (2) variants identified as intronic, intergenic, and none coding or synonymous were discarded, (3) assuming that causal variants are rare, we removed all variants with an allele frequency > 1% either in Exac [22], 1000 genomes [23] or ESP6500 (http://evs.gs.washington.edu/EVS/), (4) benign or tolerated variants, according to different in silico prediction tools were also removed. Finally, significant candidate variants were obtained after filtering against their phenotypic relevance.

### Sanger sequencing

The Sanger sequencing technique was first used to test the *BRCA* status of affected family members, then to validate the identified variants resulting from the whole exome sequencing. PCR reactions were performed on genomic DNA (gDNA), following standard protocols, followed by Sanger sequencing using an automated sequencer (ABI 3500; Applied Biosystems, Foster City, CA) using a cycle sequencing reaction kit (Big Dye Terminator kit, Applied Biosystems). Data were analyzed using BioEdit Sequence Alignment Editor Version 7.2.5.

### In silico prediction tools

We selected four in silico prediction tools to assess the functional effects of the candidate variants: Sorting Intolerant From Tolerant (SIFT) (http://sift.jcvi.org/) to examine the degree of conservation for amino acid residues across species and to find changes in protein structure and function; PolyPhen-2 (http://genetics.bwh.harvard.edu/pph2/) and Mutation Taster (http://www.mutationtaster.org/) to assess the impact of mutations on protein function and to look at effects on splicing or mRNA expression and Align GVGD (http://agvgd.iarc.fr) that classifies missense variants in a query sequence into seven grades, from the most deleterious C65 to the least deleterious C0, with the intermediate grades C15, C25, C35, C45 and C55 [24]. The program is based on Grantham calculation, a combination of Grantham Variation (GV) which measures the amount of observed biochemical evolutionary variation at a specific position of the alignment, and Grantham Deviation (GD) which measures the biochemical difference between the missense residue and the range of variation observed at this position in the alignment.

### Functional annotation and biological network construction

To discern the implication of the candidate breast cancer genes, several bioinformatics tools have been used to explore their biological pathways and the possible protein–protein interactions.

We first performed a functional analysis using the EnrichR platform [25], a bioinformatics web-based tool that includes more than 60 gene-set libraries, such as Gene ontology [26], KEGG, Wikipathways, as well as Jensen-diseases. The selection criteria for significantly enriched pathways and ontology term were a *p* value less than 0.05 (Additional file 1: Table S1).

For a better visualization and interpretation of the biological processes associated with selected breast cancer candidate genes and their upstream regulator, we used ClueGO [27], a user friendly Cytoscape plug-into analyze interrelations of terms and functional groups in biological networks [28]. In brief, we used enrichment (right-sided) hyper-geometric distribution tests, with a p value significance level ≤ 0.05, followed by the Bonferroni adjustment for the terms and the groups with Kappa-statistics score threshold set to 0.5, and leading term groups were selected based on the highest significance.

Protein–protein interaction network including physical and functional association across our set of genes was sorted out using string db 10.0 [29] with confidence score 0.4.

## Results

Eight affected individuals from seven BRCAx Tunisian families at high risk of breast cancer were analyzed using whole exome sequencing. Results including number of reads, sample coverage and sequencing depth of the whole exome sequenced patients have been summarized in Additional file 1: Table S2.

**Fig. 1:**
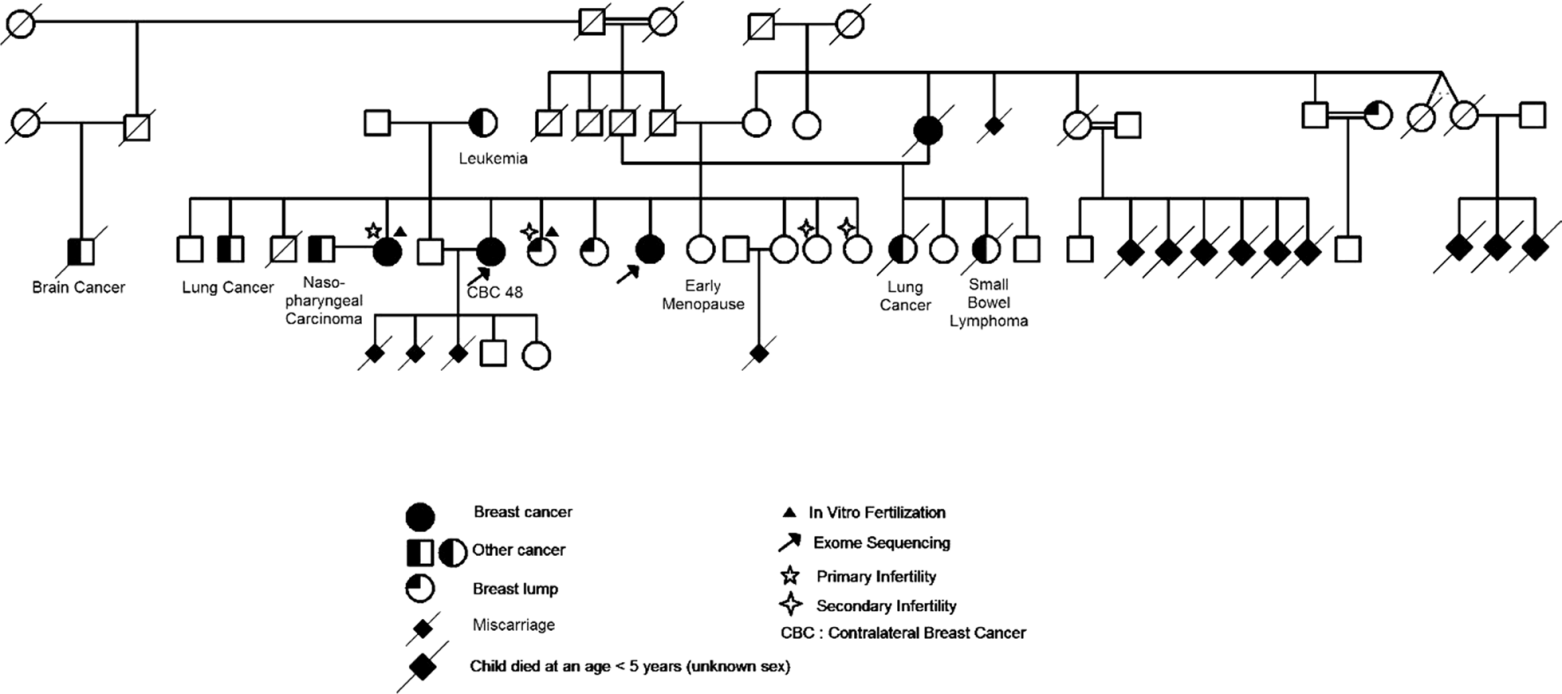
The familial pedigree of the breast cancer whole exome sequenced family

We focused our current analysis on the first *BRCA* negative family; BC-TN-F001 (Fig. 1). Two out of three affected family members have been selected for whole exome sequencing.

### Analysis of variants located on the known breast cancer susceptibility genes

Before applying the filter, steps described in the methods section, we first investigated the following 29 genes known to be associated with hereditary breast and ovarian cancer*: ATM, BARD1, BRCA1, BRCA2, BLM, BRIP1, CDH1, CHEK2, FAM175A, FANCC, FANCM, MAPKAP1, MLH1, MRE11A, MSH2, NBN, NF1, PALB2, PMS2, PTEN, RAD50, RAD51B, RAD51C, RAD51D, RECQL, RINT1, STK11, TP53* and *XRCC2* (Table 2). 59 shared heterozygous variants have been identified on these genes of which, 51 (86.4%) common non-coding variants, five exonic variants and 3 splicing SNPs. The exonic variations include a *BRCA2* rare variant (rs4987047, MAF = 0.0089), three common exonic polymorphisms on *BARD1* (rs2070094, rs2229571 and rs1048108), and one variant on *MAPKAP1* (rs1201689). None of the heterozygous variants that have been found on *BRCA1, BLM, FAM175A, FANCM, PTEN, RAD50, RINT1, STK11, TP53* and *XRCC2* were shared between the two sequenced family members.

**Table 2:** Variants on hereditary breast and ovarian cancer genes shared by the two sequenced family members

Genes	Position	Variant ID	Sequence variation	Frequency (1000 genomes)	Localization	ClinVar
***ATM***	108137775	rs642496	c.2467−123T > A	0.681909	Intronic	–
	108225661	rs664143	c.640+30986T>C	0.628195	Intronic	–
	10815,707	rs3218681	c.3403−15_3403−14insA	0.542133	Intronic	Benign
***BARD1***	215632255	rs2070094	c.1462G>A	0.366214	Exonic	Likely benign
	215645464	rs2229571	c.1077G>C	0.459265	Exonic	Likely benign
	215674224	rs1048108	c.70C>T	0.33127	Exonic	Likely benign
	215632155	rs5031009	c.1568+51A>G	0.366214	Intronic	–
	215632126	rs398048293	c.1511+78_1511+79delAA	0.366214	Intronic	–
	215634055	rs6704780	c.1315−19G>A	0.365216	Intronic	Benign
	21532192	rs5031011	c.1568+14C>T	0.352236	Intronic	Likely benign
	215595645	rs16852600	c.1904−413G>A	0.275359	Intronic	–
***BLM***	No detected variants					
***BRCA1***	No detected variants					
***BRCA2***	32953529	rs4987047	c.8830A>T	0.00898562	Exonic	Benign
***BRIP1***	No detected variants					
***CDH1***	68857277	rs201760019	c.1754−25C>A	0.000599042	Intronic	–
	68857544	rs34939176	c.1981+17_1981+18insA	0.0459265	Intronic	Benign
	68868148	rs140240766	c.*746C>A	0.000599042	UTR3	Likely benign
***CHEK2***	29137944	rs2236142	c.−194C>G	0.560304	Upstream	–
***FAM175A***	No detected variants					
***FANCC***	9,873957	rs4647534	c.1155−38T>C	0.541334	Intronic	Benign
	97873435	rs2404457	c.1329+310C>T	0.411142	UTR3	–
	97888730	rs4647512	c.896+81G>A	0.0313498	Intronic	–
***FANCM***	No detected variants					
***MAPKAP1***	128321827	rs146481224	c.848+85T>A	0.0163738	Intronic	–
	42103822	rs1197672	c.328−333C>T	0.239816	Intronic	–
	42105918	rs1201689	c.937C>G	0.305112	Exonic	–
	42111933	rs890497	c.2499+85G>A	0.0884585	Intronic	–
***MLH1***	37070437	rs41562513	c.1558+14G>A	0.0501198	Intronic	Benign
***MRE11A***	94179125	rs1014666	c.1784−69A>G	0.517173	Intronic	–
	94212048	rs535801	c.403−6G>A	0.313099	Splicing	Benign
	94197568	rs640627	c.1099−163G>A	0.314896	Intronic	–
	94225807	rs496797	c.20+141G>A	0.552915	Splicing	–
	94225920	rs497763	c.20+28G>A	0.457268	Intronic	Benign
	94212154	rs680695	c.403−112T>C	0.313099	Intronic	–
***MSH2***	47656801	rs2347794	c.1077−80G>A	0.59365	Intronic	Benign
	47630550	rs2303426	c.211+9C>A	0.628395	Intronic	Benign
	47693959	rs3732183	c.1661+12G>A	0.483427	Intronic	Benign
	47693706	rs3732182	c.1511−91G>T	0.483027	Intronic	Benign
	47739551	rs2303424	c.2744A>G	0.527955	Intergenic	–
***NBN***	90983317	rs104895036	c.456+84G>C	0.00139776	Intronic	–
***NF1***	29685905	rs34513299	c.8051−82A>G	0.00199681	Intronic	–
***PALB2***	23640467	rs249954	c.2586+58C>T	0.35004	Intronic	Benign
	23652525	rs8053188	c.−339C>T	0.0662939	UTR5	Benign
***PMS2***	6037058	rs549498051	c.706−5delT	0.453075	Splicing	Benign
***PTEN***	No detected variants					
***RAD50***	131927748	rs10520116	c.1793+22T>C	0.0129792	Intronic	–
	131944964	rs2066742	c.2923−11_2923−10insT	0.0734824	Intronic	Likely benign
	131928652	rs2706366	c.1793+926A>G	0.123003	Intronic	–
	131892979	rs4526098	c.−38A>G	0.92492	UTR5	Benign
***RAD51B***	68290372	rs17783124	c.84+28T>G	0.250399	Intronic	–
	68290464	rs28623567	c.84+120G>A	0.2498	Intronic	–
	68937054	rs142879847	c.1036+2087A>G	0.00798722	Intronic	–
	68758575	rs10129646	c.757−26T>C	0.138379	Intronic	–
	68301767	rs34564590	c.199−29_199−28insA	0.319489	Intronic	–
	68290426	rs28604984	c.84+82T>C	0.2498	Intronic	–
	68934860	rs34436700	c.958−29A>G	0.00778754	Intronic	Likely benign
	69117512	rs8023214	c.1037−32142T>C	0.528554	Intergenic	–
	69117387	rs8021657	c.1037−32267A>G	0.527556	Intergenic	–
***RAD51C***	56798207	rs28363318	c.904+34T>C	0.205272	Intronic	–
	56769979	rs12946397	c.−681G>A	0.158347	UTR5	Likely benign
***RAD51D***	No detected variants					
***RECQL***	21629993	rs397718052	c.868−68_868−67insG	0.488818	Intronic	–
	21628320	rs10841831	c.1216+82G>A	0.486821	Intronic	–
	21628791	rs3752648	c.950−33A>G	0.48742	Intronic	–
	21628336	rs10841832	c.1216+66C>T	0.486821	Intronic	–
***RINT1***	No detected variants					
***STK11***	No detected variants					
***TP53***	No detected variants					
***XRCC2***	No detected variants					

Based on breast cancer information core (BIC) and ClinVar databases, none of the 59 variants identified on these classical breast and ovarian cancer genes was classified as pathogenic. Thus, we suggested that breast cancer genetic predisposition in this family might be due to new variants on novel breast cancer candidate genes.

### Identification of novel candidate variants

**Fig. 2:**
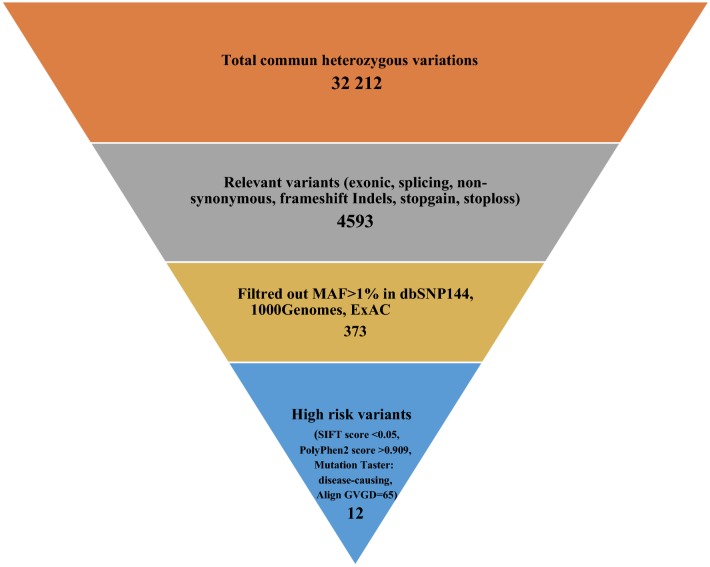
Number of variants filtered using several criteria determining high risk alleles

A total of 32,212 heterozygous variants shared by both cases have been identified (Fig. 2). Among them, 4593 heterozygous, exonic, splicing and non-synonymous SNPs were called. Variants with MAF > 1% have been excluded. Therefore, 373 rare variations have been selected for further investigations including 39 variations that have not been previously reported. In fact, as the Tunisian population is not represented in public databases, reported variants have not been excluded.

In order to select the most relevant SNPs, SIFT (score < 0.05), PolyPhen (score > 0.909), Mutation Taster (disease-causing prediction) and Align GVGD (score > C55) have been used as in silico prediction tools to assess the functional effect of the 373 variants.

A list of 12 high risk variants have been selected based on interesting in silico predictions (Table 3) of which seven nonsynonymous variants on *HSD3B1*, *PBK, ITIH2, MMS19, PPL, DNAH3 and RASSF2*, 1 splicing variation on *CFTR*, 2 stop-gain variants on *CALCOCO2* and *LRRC29*, 1 frameshift deletion on *PABPC3* and 1 frameshift insertion on *ZNF677*. None of these variants have been listed in the ClinVar database, except CFTR-rs1057516216 variant that seems to be “likely pathogenic”.

**Table 3:** Damaging variations identified in the affected individuals and selected using different functional prediction tools

Chromosome-Position^a^	Locus	Gene	Reference sequence	Variant type	Coding change	Protein variation	Variant Id	Frequency	Prediction of variant effect				Conservation score PhastCons^b^	ClinVar
							dbSNP	ExAC	SIFT	Polyphen2	Mutation taster	Align-GVGD		
Chr1: 120056630	1p12	*HSD3B1*	NM_000862	Nonsynonymous	c.484G > T	p.A162S	rs997216232	N/A	Damaging	Probably Damaging	Disease causing	C65	0.995	N/A
Chr7:117232713	7q31	*CFTR*	NM_000492	Splicing	c.2490 + 2T > C	–	rs1057516216	N/A	–	–	Disease causing	–	0.998	Likely Pathogenic
Chr8: 27668533	8p21	*PBK*	NM_018492	Nonsynonymous	c.714G > C	p.W238C	rs774498834	8.265e−06	Damaging	Probably Damaging	Disease causing	C65	1	N/A
Chr10:7751028	10p14	*ITIH2*	NM_002216	Nonsynonymous	c.236C > A	p.S79Y	rs749149620	9.884e−05	Damaging	Probably Damaging	Disease causing	C65	1	N/A
Chr10:99238117	10q24	*MMS19*	NM_001289403	Nonsynonymous	c.292C > T	p.R98W	rs29001280	0.0015	Damaging	Probably Damaging	Disease causing	C65	1	N/A
Chr13:25671311	13q12	*PABPC3*	NM_030979	Frameshift deletion	c.975_979del	p V325 fs	rs371130768	8.237e−06	–	–	Disease causing	–	1	N/A
Chr16:4934532	16p13	*PPL*	NM_002705	Nonsynonymous	c.4124T > G	p.I1375S	N/A	N/A	Damaging	Probably Damaging	Disease causing	C65	1	N/A
Chr16:21011744	16p12	*DNAH3*	NM_017539	Nonsynonymous	c.6223C > T	p.P2075S	N/A	N/A	Damaging	Probably Damaging	Disease causing	C65	1	N/A
Chr16:67241867	16q22	*LRRC29*	NM_001004055	Stopgain	c.412C > T	p.R138X	rs776721799	8.582e−06	–	–	Disease causing	–	0.259	N/A
Chr17:46940292	17q21	*CALCOCO2*	NM_005831	Stopgain	c.1266T > A	p.C422X	N/A	N/A	–	–	Disease causing	–	0.999	N/A
Chr19: 53740406	19q13	*ZNF677*	NM_182609	Frameshift insertion	c.1573dupA	p.T525 fs	rs566714089	0.0038	–	–	Disease causing	–	–	N/A
Chr20: 4766902	20p13	*RASSF2*	NM_170774	Nonsynonymous	c.886C > T	p.R296 W	rs756486184	8.238e−06	Damaging	Probably Damaging	Disease causing	C65	0.998	N/A

^a^GRCh37/hg19; ^b^ PhastCons values vary between 0 and 1 and reflect the probability that each nucleotide belongs to a conserved element, based on the multiple alignment of genome sequences of 46 different species (the closer the value is to 1, the more probable the nucleotide is conserved)

### The family specific hypothesis

We first filtered this list of candidate genes and variants against the additional six BRCAx exome sequenced breast cancer families (BC-TN-F002_BC-TN-F007). All identified variants have been only found in BC-TN-F001, expect the *PABPC3* variant that was found in other Tunisian BRCAx families.

Then, we compared the list of variants identified in this family to results from other WES studies on BRCAx families. Again, variants identified in this study were only found in BC-TN-F001, suggesting a family specific predisposition to breast cancer. This family specific hypothesis has been suggested to explain the breast cancer predisposition in 4 other WES studies [8, 19–21].

We therefore performed a literature curation based on the results of the 4 family specific WES studies and the current one in order to explore this family specific predisposition to breast cancer. Additional file 1: Table S3 summarizes the list of 54 genes identified through these studies as new potential breast cancer candidate genes inherited in a family specific model. We observed that each exome sequenced family showed a specific genetic pattern with a different set of candidate genes. Only *KAT6B* has been reported in two different families from two separate studies [19, 20].

In a recent WES study performed on five BRCAx Egyptian families [8], four genes namely *LOC100129697*, *NPIPB1, NBPF10* and *PABPC3* have been identified in more than one family. *PABPC3* is also found to be shared between three Egyptian families and the four Tunisian families sequenced in this current study.

### Gene set enrichment analysis

As most of the breast cancer candidate genes identified through family specific predisposition studies lack functional evidence of their involvement in breast carcinogenesis, we pooled the 54 candidate genes identified in separate WES studies (Additional file 1: Table S3) and we performed functional annotation analysis to explore if there is any biological interaction between these genes which may strengthen their association with breast cancer (Additional file1: Table S1; Additional file 2: Figure S1).

**Fig. 3:**
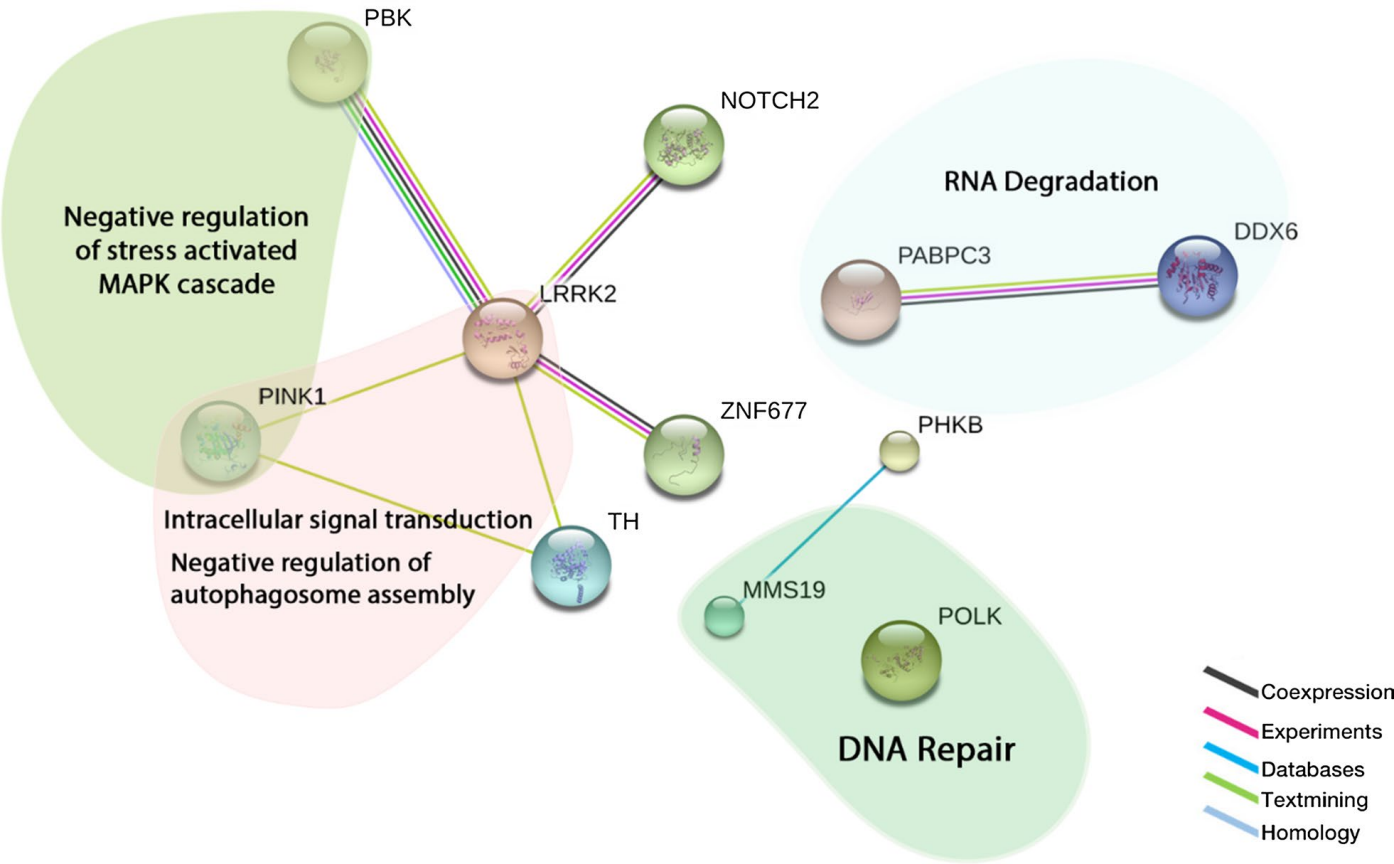
Protein-Protein interactions of novel breast cancer candidate genes identified in four WES breast cancer studies. Genes are clustered in four pathways related to cancer etiology. The lines represent the levels of evidence as indicated in the color legend

Moreover, a comprehensive gene set enrichment combined with a protein–protein interaction analysis was performed using both of EnrichR and Stringdb webtools. Results showed that *MMS19* and *POLK* genes are involved in the DNA repair pathway (Fig. 3). The remaining genes are a part of several pathways involved in cancer etiology such as: Negative regulation of stress activated MAPK cascade (*PBK* and *PINK1*), intracellular signal transduction and regulation of autophagosome assembly (*LRRK2* and *PINK1*) and RNA degradation (*PABC3* and *DDX6*). *NOTCH2* and *ZNF677* are highly predicted to be co-expressed with *PBK* and *LRRK2* (Fig. 3).

**Fig. 4:**
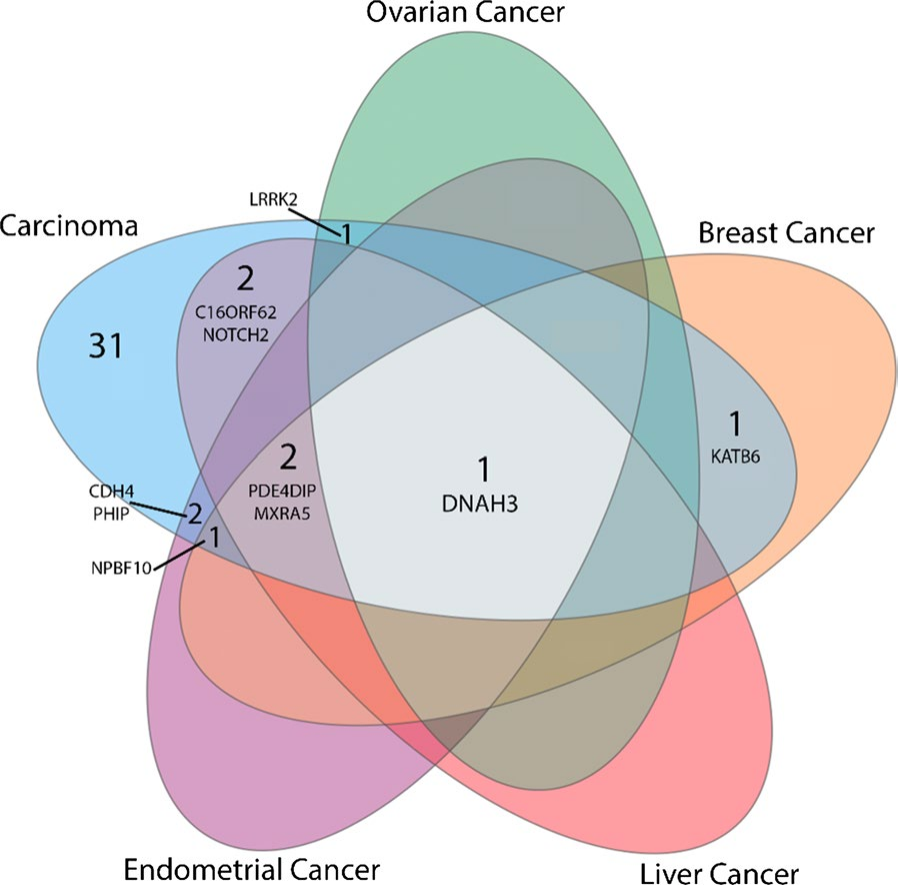
Venn diagram representing the involvement of the identified breast cancer candidate genes in several cancers

Finally, we performed a disease genes association analysis using Jensen disease database (PMID: 25484339) by clustering the candidates genes into subgroups involved in a same disease. We, therefore, examined the overlap between these sub-clusters and different cancers namely, breast, ovarian, liver and endometrial cancers (Fig. 4). The results obtained show five top significant genes involved in breast cancers that are *DNHA3, KATB6, PDE4DIP, MXRA5* and *NBPF10*. Of note, *NBPF10* is also linked to endometrial cancer and *DNHA3* is the only candidate that is involved in all these cancers.

## Discussion

The majority of BRCAx patients with familial breast cancer lack evidence for their genetic predisposition. Multiple models have been proposed to explain the missing heritability. First, recessive and polygenic models of transmission have been proposed to resolve a part of breast cancer remaining heritability [30]. Another class of genetic variations that contributes to familial breast cancer risk includes large deletions and copy number variation [31]. Interactions between genetic variants and environmental risk factors remain an interesting model to explain breast cancer predisposition in multiple families. However, this model is largely unexplored because most of association studies that could address this model are underpowered [32]. Finally, NGS application using family-based approach represents an appropriate modality to identify additional genes with autosomal dominant mechanism of inheritance and thus explains an additional part of the breast cancer familial component [7].

In the present study, two affected sisters from a non *BRCA* Tunisian breast cancer family have been explored using whole exome sequencing. We excluded unaffected members in our sequenced individuals since they could be non-penetrant carriers.

Thousands of heterozygous variants shared between the two sequenced family members have been identified. However, no deleterious variants have been found within known breast cancer genes. *BRCA2*-rs4987047 is the only rare exonic variant identified on the known breast cancer susceptibility genes. Despite its potential functional effect [33], the ClinVar predictions classify this variant as benign.

Of note, among 108 exome sequenced families previously reported in 10 breast cancer WES studies, mutations on known breast cancer genes have been reported in only four families because BRCA tests are usually performed before using the whole exome sequencing approach [10–14]. Moreover, the high rate of consanguinity in the Tunisian population, may decrease the prevalence of breast cancer by decreasing the frequency of high penetrant mutations [34].

However, several common variants located on known breast cancer susceptibility genes have been identified in BC-TN-F001 (Table 2). Some of these variants have been previously reported as associated with different cancers as low penetrant polymorphisms. Indeed, two common exonic variants identified on *BARD1* gene (rs2229571 and rs1048108) have been identified as low penetrant breast cancer loci in the Chinese population [35]. Moreover, *PALB2*-rs249954 has been reported to be associated with breast cancer risk [36], *CHEK2*-rs2236142 is likely associated with a decreased risk of esophageal cancer and lymph node metastasis in a Chinese population [37], *RAD51C*-rs12946397 is known to be associated with the risk of head and neck cancer [38] and *ATM*-rs664143 has been reported to be associated with lung cancer [39]. Given the fact that multiple family members are affected by other cancers such as lung carcinoma and small bowel lymphoma (Fig. 1), the involvement of these variants in this family predisposition to cancer is possible. Therefore, we cannot discard the polygenic model of breast cancer predisposition in this Tunisian breast cancer family.

Despite the fact that these variants have been reported as common low penetrant variants in Caucasians, we cannot estimate their penetrance in the Tunisian population. Indeed, because of different genetic architectures and differences in allele frequencies between populations, variant penetrance may differ from one population to another and a low penetrant variant in one population may be of high penetrance in another population. Further association studies in large Tunisian cohorts are needed to assess the penetrance of these variants in the Tunisian population.

After investigating known breast cancer genes, we explored other genes not yet reported as associated with the breast disease. Twelve high risk variants, predicted as deleterious by four different in silico prediction tools and showing a phenotypic relevance have been selected on the following genes: *HSD3B1, CFTR, PBK, ITIH2, MMS19, PABPC3, PPL, DNAH3, LRRC29, CALCOCO2, ZNF677* and *RASSF2.*

None of the variants identified within these genes have been listed in the ClinVar database, except for the *CFTR*-rs1057516216 variant that seems to be “likely pathogenic”. *CFTR* (Cystic Fibrosis Transmembrane Conductance Regulator) is a gene that encodes a member of the ATP-binding cassette (ABC) transporter superfamily [40]. Mutations in this gene cause cystic fibrosis, the most common lethal genetic disorder in populations of Northern European descent [41]. However, *CFTR* is potentially recurrently mutated by chance because of its large size and its involvement in breast carcinogenesis is controversial, thus, it cannot be considered as a potential breast cancer candidate gene. Indeed, it has been proposed that a *CFTR* mutation may protect against breast cancer [42], however, in another study that correlated the expression level of CFTR and breast cancer histological grading, it was shown that high serum levels of CFTR were associated with a high grade and poorly differentiated tumors [43].

When comparing the identified set of genes with other genes reported in other breast cancer WES studies, we showed that each exome sequenced family has a specific genetic pattern with a different set of candidate genes. Except *PABPC3*, genes identified in this Tunisian breast cancer family have not been reported in other breast cancer exome sequenced families, suggesting a family specific genetic predisposition to the disease. *PABPC3* was shared between four Tunisian families and three Egyptian whole exome sequenced families. Moreover, *LOC100129697*, *NPIPB1, NBPF10* have been found in three whole exome sequenced Egyptian families [8]. These genes shared between families from a particular ethnic group (Tunisians and Egyptians) suggest that in populations with high consanguinity and endogamy rates, the ethnic specific breast cancer predisposition model is also plausible. PABPC3 acts in a cytoplasmic regulatory processes of mRNA metabolism [44]. The involvement of *PABPC3* in the RNA degradation pathway has been confirmed by the analysis of the biological process and protein–protein networks that we performed in this study (Additional file 2: Figure S1, Fig. 3).

We also showed that the remaining genes are also linked to interesting new pathways such as: negative regulation of stress activated MAPK cascade and intracellular signal transduction and regulation of autophagosome assembly. Only two genes (*MMS19* and *POLK*) are involved in DNA repair pathway, considered as the traditional pathway in which breast cancer genes are involved [45].

MMS19 acts as an adapter between early-acting cytosolic iron-sulfur assembly components and a subset of cellular target iron-sulfur proteins such as ERCC2/XPD, FANCJ and RTEL1, thereby playing a key role in nucleotide excision repair (NER) and RNA polymerase II (POL II) transcription [46]. Of note, the human MMS19 also interacts with estrogen receptors in a ligand-independent manner [47]. *POLK* is a member of Y family DNA polymerases, and functions by repairing the replication fork passing through DNA lesions [48]. Recently, *POLK* have been reported as a new ovarian cancer susceptibility gene [49].

Additional functional annotation analysis using the Jensen disease library, showed that the top significant genes involved in breast cancer are *KATB6, PDE4DIP, MXRA5, DNHA3* and *NBPF10*. KAT6B—a histone acetyl transferase involved in DNA replication, gene expression and regulation, and epigenetic modification of chromosomal structure [50] has been reported as associated with breast cancer in two separate WES studies [19, 20].

Consistently with our results, it has been reported that *DNHA3* is involved in different cancers including breast cancer [51–53]. *DNHA3* (Dynein Axonemal Heavy Chain 3) gene belongs to the dynein family, whose members encode large proteins that are constituents of the microtubule-associated motor protein complex [54]. Among its related pathways we denotes the respiratory electron transport, ATP synthesis by chemiosmosis coupling, and heat production by uncoupling proteins. However, little evidence exist on the roles of *PDE4DIP*, *MXRA5*, and *NBPF10* in breast carcinogenesis.

In summary, these WES studies results and the functional annotation performed in the present study, altogether showed that *MMS19, DNHA3, POLK* and *KATB6* are interesting breast cancer candidate genes. Variants located on these genes seem to be inherited in a family specific model. *PABPC3* seems to be another interesting breast cancer candidate gene that may be associated with breast cancer in an ethnic specific manner as it has been reported in another North African population [8].

Although NGS represents an unprecedented approach to decipher the genetic predisposition to different hereditary diseases, it comes with numerous challenges. Indeed, the different lists of genes that resulted from different breast cancer WES studies may be explained in part by the different pipelines and bioinformatics tools used to analyze these data. In addition, NGS data users apply different filters to help prioritize variants such as the in silico prediction tools that may mis-classify some variants and thus causes erroneous inclusion or exclusion of some variations.

Therefore, in order to assess how much the family specific hypothesis is plausible, we suggest to pool raw data from all breast cancer whole exome sequenced families and re-analyze the resulting data using a common and consensual strategy. Efforts made by the COMPLEXO group in identifying the missing breast cancer heritability via Next generation collaborations represent an excellent initiative to overcome these NGS data analysis challenges [55].

## Conclusions

In the present study we reported a list of new breast cancer candidate genes that seem to be inherited in a family specific and ethnic specific models. Further WES studies on BRCAx Tunisian families and further in vitro or in vivo functional assays are needed to understand their effects and to confirm their association with breast cancer risk. For a better interpretation of NGS data, the scientific community should first overcome NGS data analysis challenges in order to generate more meaningful NGS data and more clinically actionable variants.

## Additional files

**Additional file 1: Table S1.** Gene set enrichment analysis. **Table S2.** Summary of SNPs and Indels identified in the 7 BRCAx sequenced Tunisian breast cancer families. **Table S3.** Putative predisposition family-specific genes in several WES studies using the family-based approach.

**Additional file 2: Figure S1.** Biological networks and Enriched gene ontology pathways identified by the functional annotation analysis. Enrichment network of the shared candidate disease genes and their upstream regulator based on biological processes using ClueGO Cytoscape plugin. Hyper-geometric (right-handed) enrichment distribution tests, with a p-value significance level of ≤ 0.05, followed by the Bonferroni adjustment for the terms and leading term groups were selected based on the highest significance. The node size and deeper color indicates greater significance of the enrichment.

## Abbreviations

ABC: ATP-binding cassette
BAM: binary alignment map
BIC: Breast Cancer Information Core
BRCAx: non BRCA
BWA: Burrows–Wheeler Aligner
DNA: DeoxyriboNucleic Acid
GD: Grantham deviation
gDNA: genomic DNA
GV: Grantham variation
INDEL: insertion-deletion
MAF: Minor Allele Frequency
mRNA: Messenger RNA
NER: nucleotide excision repair
NGS: next generation sequencing
PCR: polymerase chain reaction
POL II: RNA polymerase II
RNA: ribonucleic acid
SAM: sequence alignment map
SIFT: Sorting Intolerant From Tolerant
SNP: single nucleotide polymorphism
VarAFT: Variant Annotation and Filtering Tool
WES: whole exome sequencing
