# Supplementary material for: Family specific genetic predisposition to breast cancer: results from Tunisian whole exome sequenced breast cancer cases
Source: J Transl Med. 2018 Jun 7;16:158. doi: 10.1186/s12967-018-1504-9 (PMC5992876; doi:10.1186/s12967-018-1504-9)
Supplement: Supplementary file 1 — Additional file 1: Table S1. Gene set enrichment analysis. Table S2. Summary of SNPs and Indels identified in the 7 BRCAx sequenced Tunisian breast cancer families. Table S3. Putative predisposition family-specific genes in several WES studies using the family-based approach. [file 12967_2018_1504_MOESM1_ESM.docx]

**Table S1. Gene set enrichment analysis.**

| **Term** | **P-value** | **Adjusted P-value** | **Old P-value** | **Old Adjusted P-value** | **Z-score** | **Combined Score** | **Genes** |
| --- | --- | --- | --- | --- | --- | --- | --- |
| negative regulation of autophagosome assembly (GO:1902902) | 0,000353888 | 0,035388755 | 0,000450093 | 0,045009251 | -3,583140673 | 11,97256712 | PINK1;LRRK2 |
| negative regulation of macroautophagy (GO:0016242) | 0,000283629 | 0,035388755 | 0,000375709 | 0,045009251 | -2,414873766 | 8,068965435 | PINK1;LRRK2 |
| intracellular signal transduction (GO:0035556) | 0,004715177 | 0,115293372 | 0,003810236 | 0,115013177 | -3,954984134 | 8,543854687 | GRIP1;PINK1;LRRK2; |
| positive regulation of protein binding (GO:0032092) | 0,005252724 | 0,115293372 | 0,005074991 | 0,115013177 | -2,513118316 | 5,429027519 | LRRK2;SPPL3 |
| positive regulation of protein ubiquitination (GO:0031398) | 0,008955265 | 0,115293372 | 0,008455945 | 0,115013177 | -2,543023941 | 5,493631905 | PINK1;LRRK2 |
| animal organ morphogenesis (GO:0009887) | 0,013526011 | 0,115293372 | 0,01260098 | 0,115013177 | -2,631791941 | 5,685395226 | NOTCH2;TH |
| dopamine biosynthetic process (GO:0042416) | 0,014170415 | 0,115293372 | 0,01591479 | 0,115013177 | 0,043355498 | -0,093659814 | TH |
| olfactory bulb development (GO:0021772) | 0,014170415 | 0,115293372 | 0,01591479 | 0,115013177 | 0,30072153 | -0,649641304 | LRRK2 |
| regulation of mitochondrial fission (GO:0090140) | 0,014170415 | 0,115293372 | 0,01591479 | 0,115013177 | 0,354601011 | -0,766035819 | LRRK2 |
| peptidyl-serine autophosphorylation (GO:0036289) | 0,014170415 | 0,115293372 | 0,01591479 | 0,115013177 | 0,800741046 | -1,729821133 | PINK1 |
| catecholamine biosynthetic process (GO:0042423) | 0,014170415 | 0,115293372 | 0,01591479 | 0,115013177 | 1,376531103 | -2,973686193 | TH |
| mitochondrion organization (GO:0007005) | 0,014797184 | 0,115293372 | 0,013750833 | 0,115013177 | -2,616615039 | 5,652608937 | PINK1;LRRK2 |
| GTP metabolic process (GO:0046039) | 0,016980732 | 0,115293372 | 0,018543185 | 0,115013177 | 0,16961965 | -0,366425146 | LRRK2 |
| mitochondrion localization (GO:0051646) | 0,016980732 | 0,115293372 | 0,018543185 | 0,115013177 | 0,504437714 | -1,089724352 | LRRK2 |
| positive regulation of ATP biosynthetic process (GO:2001171) | 0,016980732 | 0,115293372 | 0,018543185 | 0,115013177 | 0,818240358 | -1,767624467 | PINK1 |
| regulation of protein targeting to mitochondrion (GO:1903214) | 0,016980732 | 0,115293372 | 0,018543185 | 0,115013177 | 1,542115847 | -3,331394832 | PINK1 |
| positive regulation of autophagosome maturation (GO:1901098) | 0,016980732 | 0,115293372 | 0,018543185 | 0,115013177 | 2,233353709 | -4,82465894 | CALCOCO2 |
| eye photoreceptor cell development (GO:0042462) | 0,016980732 | 0,115293372 | 0,018543185 | 0,115013177 | 2,515268221 | -5,433671906 | TH |
| norepinephrine biosynthetic process (GO:0042421) | 0,016980732 | 0,115293372 | 0,018543185 | 0,115013177 | 3,986367743 | -8,611651924 | TH |
| positive regulation of insulin-like growth factor receptor signaling pathway (GO:0043568) | 0,019783178 | 0,115293372 | 0,021164749 | 0,115013177 | -0,240431742 | 0,519398763 | PHIP |
| neuron death (GO:0070997) | 0,019783178 | 0,115293372 | 0,021164749 | 0,115013177 | -0,0789034 | 0,170453068 | LRRK2 |
| positive regulation of calcineurin-NFAT signaling cascade (GO:0070886) | 0,019783178 | 0,115293372 | 0,021164749 | 0,115013177 | 2,265007841 | -4,89304058 | SPPL3 |
| marginal zone B cell differentiation (GO:0002315) | 0,019783178 | 0,115293372 | 0,021164749 | 0,115013177 | 2,293249793 | -4,954050971 | NOTCH2 |
| cellular response to oxidative stress (GO:0034599) | 0,02036829 | 0,115293372 | 0,0187823 | 0,115013177 | -2,149623504 | 4,643778643 | PINK1;LRRK2 |
| positive regulation of programmed cell death (GO:0043068) | 0,022577774 | 0,115293372 | 0,023779499 | 0,115013177 | -0,337986388 | 0,730143658 | LRRK2 |
| negative regulation of protein processing (GO:0010955) | 0,022577774 | 0,115293372 | 0,023779499 | 0,115013177 | -0,336077329 | 0,726019566 | LRRK2 |
| atrial septum morphogenesis (GO:0060413) | 0,022577774 | 0,115293372 | 0,023779499 | 0,115013177 | -0,283583916 | 0,61261934 | NOTCH2 |
| histone acetylation (GO:0016573) | 0,022577774 | 0,115293372 | 0,023779499 | 0,115013177 | 0,239627388 | -0,517661137 | KAT6B |
| positive regulation of cytosolic calcium ion concentration (GO:0007204) | 0,022909967 | 0,115293372 | 0,021075195 | 0,115013177 | -2,08244052 | 4,498644899 | XCR1;SPPL3 |
| Notch signaling involved in heart development (GO:0061314) | 0,025364542 | 0,115293372 | 0,026387453 | 0,115013177 | -0,411725672 | 0,889440815 | NOTCH2 |
| regulation of synaptic vesicle exocytosis (GO:2000300) | 0,025364542 | 0,115293372 | 0,026387453 | 0,115013177 | -0,387148335 | 0,836346999 | LRRK2 |
| synaptic transmission, dopaminergic (GO:0001963) | 0,025364542 | 0,115293372 | 0,026387453 | 0,115013177 | 0,164042457 | -0,354376875 | TH |
| membrane protein proteolysis (GO:0033619) | 0,025364542 | 0,115293372 | 0,026387453 | 0,115013177 | 0,645158141 | -1,39371922 | SPPL3 |
| cellular response to dopamine (GO:1903351) | 0,025364542 | 0,115293372 | 0,026387453 | 0,115013177 | 1,699257542 | -3,670864161 | LRRK2 |
| excitatory postsynaptic potential (GO:0060079) | 0,025364542 | 0,115293372 | 0,026387453 | 0,115013177 | 2,399376228 | -5,183313292 | LRRK2 |
| negative regulation of hydrogen peroxide-induced cell death (GO:1903206) | 0,025364542 | 0,115293372 | 0,026387453 | 0,115013177 | 2,478509198 | -5,354262296 | LRRK2 |
| cell fate determination (GO:0001709) | 0,025364542 | 0,115293372 | 0,026387453 | 0,115013177 | 2,606385446 | -5,630510201 | NOTCH2 |
| regulation of dendritic spine morphogenesis (GO:0061001) | 0,025364542 | 0,115293372 | 0,026387453 | 0,115013177 | 2,951419523 | -6,375878808 | LRRK2 |
| regulation of protein kinase A signaling (GO:0010738) | 0,025364542 | 0,115293372 | 0,026387453 | 0,115013177 | 3,294187153 | -7,116351265 | LRRK2 |
| positive regulation of NFAT protein import into nucleus (GO:0051533) | 0,028143503 | 0,117264598 | 0,028988627 | 0,115013177 | 0,364140165 | -0,780469765 | SPPL3 |
| cilium-dependent cell motility (GO:0060285) | 0,028143503 | 0,117264598 | 0,028988627 | 0,115013177 | 0,499038276 | -1,069599904 | DNAH3 |
| negative regulation of reactive oxygen species metabolic process (GO:2000378) | 0,028143503 | 0,117264598 | 0,028988627 | 0,115013177 | 2,178273885 | -4,668743167 | PINK1 |
| pulmonary valve morphogenesis (GO:0003184) | 0,028143503 | 0,117264598 | 0,028988627 | 0,115013177 | 2,598637586 | -5,569718093 | NOTCH2 |
| response to ethanol (GO:0045471) | 0,03091468 | 0,120278904 | 0,031583039 | 0,115013177 | 0,124462743 | -0,263604874 | TH |
| cytoplasmic mRNA processing body assembly (GO:0033962) | 0,03091468 | 0,120278904 | 0,031583039 | 0,115013177 | 3,434177509 | -7,273388899 | DDX6 |
| nervous system development (GO:0007399) | 0,033410655 | 0,120278904 | 0,028711709 | 0,115013177 | -2,846811284 | 6,029381283 | NOTCH2;AVIL;MTR |
| glycogen metabolic process (GO:0005977) | 0,033678093 | 0,120278904 | 0,034170707 | 0,115013177 | 0,387976153 | -0,821711003 | PHKB |
| iron-sulfur cluster assembly (GO:0016226) | 0,033678093 | 0,120278904 | 0,034170707 | 0,115013177 | 0,568511324 | -1,20407403 | MMS19 |
| positive regulation of Ras protein signal transduction (GO:0046579) | 0,033678093 | 0,120278904 | 0,034170707 | 0,115013177 | 2,081155098 | -4,407765863 | NOTCH2 |
| regulation of protein ubiquitination (GO:0031396) | 0,033678093 | 0,120278904 | 0,034170707 | 0,115013177 | 2,559658864 | -5,421209102 | PINK1 |
| neuromuscular junction development (GO:0007528) | 0,033678093 | 0,120278904 | 0,034170707 | 0,115013177 | 3,257356556 | -6,89889237 | LRRK2 |
| regulation of reactive oxygen species metabolic process (GO:2000377) | 0,036433764 | 0,120407444 | 0,036751646 | 0,115013177 | 2,160969014 | -4,574498948 | PINK1 |
| positive regulation of mitochondrial fission (GO:0090141) | 0,036433764 | 0,120407444 | 0,036751646 | 0,115013177 | 2,383731847 | -5,04605978 | PINK1 |
| regulation of mitochondrion organization (GO:0010821) | 0,036433764 | 0,120407444 | 0,036751646 | 0,115013177 | 2,604257031 | -5,512883791 | PINK1 |
| cellular protein complex assembly (GO:0043623) | 0,039181714 | 0,120407444 | 0,039325874 | 0,115013177 | 1,784956738 | -3,778528366 | PDE4DIP |
| negative regulation of endoplasmic reticulum stress-induced intrinsic apoptotic signaling pathway (GO:1902236) | 0,039181714 | 0,120407444 | 0,039325874 | 0,115013177 | 2,016706845 | -4,269114124 | LRRK2 |
| Golgi to plasma membrane transport (GO:0006893) | 0,039181714 | 0,120407444 | 0,039325874 | 0,115013177 | 2,437172558 | -5,159187027 | C16ORF62 |
| positive regulation of protein phosphorylation (GO:0001934) | 0,041283779 | 0,120407444 | 0,037643127 | 0,115013177 | -1,708197332 | 3,616038383 | PINK1;LRRK2 |
| preassembly of GPI anchor in ER membrane (GO:0016254) | 0,041921964 | 0,120407444 | 0,041893408 | 0,115013177 | 0,654114126 | -1,384677134 | PIGN |
| positive regulation of protein dephosphorylation (GO:0035307) | 0,041921964 | 0,120407444 | 0,041893408 | 0,115013177 | 0,689858057 | -1,46034253 | SPPL3 |
| regulation of proteasomal protein catabolic process (GO:0061136) | 0,041921964 | 0,120407444 | 0,041893408 | 0,115013177 | 2,226569817 | -4,713367577 | PINK1 |
| regulation of protein complex assembly (GO:0043254) | 0,041921964 | 0,120407444 | 0,041893408 | 0,115013177 | 2,564333814 | -5,428371373 | PINK1 |
| regulation of synaptic transmission, glutamatergic (GO:0051966) | 0,041921964 | 0,120407444 | 0,041893408 | 0,115013177 | 3,16885858 | -6,708074084 | LRRK2 |
| positive regulation of transcription, DNA-templated (GO:0045893) | 0,043097006 | 0,120407444 | 0,035206709 | 0,115013177 | -3,330148881 | 7,049505317 | GRIP1;KAT6B;MMS19;PHIP |
| positive regulation of protein autophosphorylation (GO:0031954) | 0,044654536 | 0,120407444 | 0,044454265 | 0,115013177 | 1,967984781 | -4,165975658 | RASSF2 |
| regulation of neuron death (GO:1901214) | 0,044654536 | 0,120407444 | 0,044454265 | 0,115013177 | 2,143097921 | -4,536668098 | LRRK2 |
| glycogen catabolic process (GO:0005980) | 0,044654536 | 0,120407444 | 0,044454265 | 0,115013177 | 2,476234943 | -5,241877172 | PHKB |
| phospholipid translocation (GO:0045332) | 0,044654536 | 0,120407444 | 0,044454265 | 0,115013177 | 2,509710172 | -5,312740011 | ATP10B |
| DNA repair (GO:0006281) | 0,046689464 | 0,120407444 | 0,042521089 | 0,115013177 | -0,645473501 | 1,36638602 | MMS19;POLK |
| regulation of canonical Wnt signaling pathway (GO:0060828) | 0,04737945 | 0,120407444 | 0,047008462 | 0,115013177 | 2,125684613 | -4,49980632 | LRRK2 |
| negative regulation of JNK cascade (GO:0046329) | 0,04737945 | 0,120407444 | 0,047008462 | 0,115013177 | 2,177181867 | -4,608819513 | PINK1 |

**Table S2. Summary of SNPs and Indels identified in the 7 BRCAx sequenced Tunisian breast cancer families**

|  | **BC-TN-F001** | | **BC-TN-F002** | **BC-TN-F003** | **BC-TN-F004** | **BC-TN-F005** | **BC-TN-F006** | **BC-TN-F007** |
| --- | --- | --- | --- | --- | --- | --- | --- | --- |
|  | **F001-1** | **F001-2** |  |  |  |  |  |  |
| **Total reads** | 52 713 285 | 62 954 053 | 49 111 123 | 56 094 160 | 65 433 181 | 50 721 434 | 53 120 773 | 52 855 775 |
| **% Reads mapped to human genome** | 99.41 | 99.40 | 99.64 | 99.46 | 99.44 | 99.46 | 99.65 | 99.64 |
| **Total coverage** | 3 276 301 158 | 3 878 573 796 | 2 718 453 424 | 3 494 380 561 | 3 954 519 193 | 3 074 996 362 | 2 909 347 349 | 2 907 656 083 |
| **Mean read depth of target regions (X)** | 63.56 | 75.25 | 52.74 | 67.80 | 76.72 | 59.66 | 56.45 | 56.41 |
| **% Coverage of target regions (more than 10X)** | 91.9 | 93.0 | 91.3 | 92.3 | 93.0 | 91.7 | 91.7 | 91.6 |
| **% Coverage of target regions (more than 20X)** | 85.3 | 88.0 | 82.7 | 92.3 | 88.1 | 84.6 | 83.9 | 83.8 |
| **Number of SNPs (Heterozygous)** | 54 916 | 62 763 | 53 480 | 53 003 | 59 153 | 51 826 | 56 836 | 55 833 |
| **Number of coding SNPs** | 14 962 | 15 126 | 14 452 | 13 877 | 15 113 | 14 053 | 15 028 | 15 051 |
| **Number of splicing SNPs** | 422 | 447 | 404 | 399 | 423 | 397 | 455 | 405 |
| **Number of synonymous SNP** | 7486 | 7679 | 7321 | 6988 | 7596 | 7078 | 7573 | 7556 |
| **Number of nonsynonymous SNPs** | 6806 | 6823 | 6445 | 6225 | 6780 | 6348 | 6788 | 6792 |
| **Number of non frameshift INDELs** | 199 | 199 | 224 | 183 | 246 | 197 | 216 | 219 |
| **Number of non frameshift substitutions** | 0 | 1 | 0 | 1 | 2 | 1 | 1 | 1 |
| **Number of frameshift INDELs** | 95 | 101 | 103 | 103 | 106 | 103 | 114 | 103 |
| **Number of stopgain SNPs** | 67 | 65 | 59 | 51 | 59 | 52 | 50 | 65 |
| **Number of stoploss SNPs** | 9 | 5 | 5 | 4 | 5 | 8 | 6 | 6 |

Family BC-TN-F001 is the non BRCA breast cancer family investigated in this study. F001-1 and F001-2 are the two affected family members that have been sequenced

**Table S3. Putative predisposition family-specific genes in several WES studies using the family-based approach**

| Reference | Current study | (Lynch et al. 2013) | (Wen et al. 2014) | | | (Noh et al. 2015) | | (C. Kim et al.2016) | | | |
| --- | --- | --- | --- | --- | --- | --- | --- | --- | --- | --- | --- |
| BRCAx Families | **Family 1** | **Family 1** | **Family 1** | **Family 2** | **Family 3** | **Family 1** | **Family 1** | **Family 2** | **Family 3** | **Family 4** | **Family 5** |
| *Candidate Genes* | *CALCOCO2* | *GBP4* | *GPRIN1* | ***KAT6B*** | *NANP* | *ACCS* | *ABCA10* | ***C16orf62*** | *CCDC7* | ***C16orf62*** | *ATP10B* |
|  | *CFTR* | *AVIL* | *PINK1* | *NOTCH2* | *PHKB* | *CCNF* | *CHST15* | *KRTAP21-3* | *CXorf23* | *GAGE2A* | ***NPIPB11*** |
|  | *DNAH3* | *C601F170* | *POLK* |  |  | *DDL1* | *GRIP1* | ***LOC100129697*** | ***LOC100129697*** | ***NPIPB11*** | *PIGN* |
|  | *HSD3B1* | *DDX6* |  |  |  | *SPPL3* | ***LOC100129697*** | ***NBPF10*** | ***NBPF10*** | *PHIP* | *PRR14L* |
|  | *ITIH2* | *DHX57* |  |  |  | *SRL* | *LOC388813* | *NPIPB13* | ***NPIPB11*** | *SLC15A5* |  |
|  | *LRRC29* | *GALNT9* |  |  |  | *TH* | ***NBPF10*** | ***PABPC3*** | ***PABPC3*** | *ZNF750* |  |
|  | *MMS19* | ***KAT6B*** |  |  |  | *XCR1* | ***PABPC3*** | *PDE4DIP* | *SMIM13* |  |  |
|  | ***PABPC3*** | *KIAA1586* |  |  |  |  |  |  |  |  |  |
|  | *PBK* | *LRRK2* |  |  |  |  |  |  |  |  |  |
|  | *PPL* | *MXRA5* |  |  |  |  |  |  |  |  |  |
|  | *RASSF2* |  |  |  |  |  |  |  |  |  |  |
|  | *ZNF677* |  |  |  |  |  |  |  |  |  |  |

*Genes in bold are those shared between more than one family
