# Supplementary figures and images for: Family specific genetic predisposition to breast cancer: results from Tunisian whole exome sequenced breast cancer cases
Source: J Transl Med. 2018 Jun 7;16:158. doi: 10.1186/s12967-018-1504-9 (PMC5992876; doi:10.1186/s12967-018-1504-9)

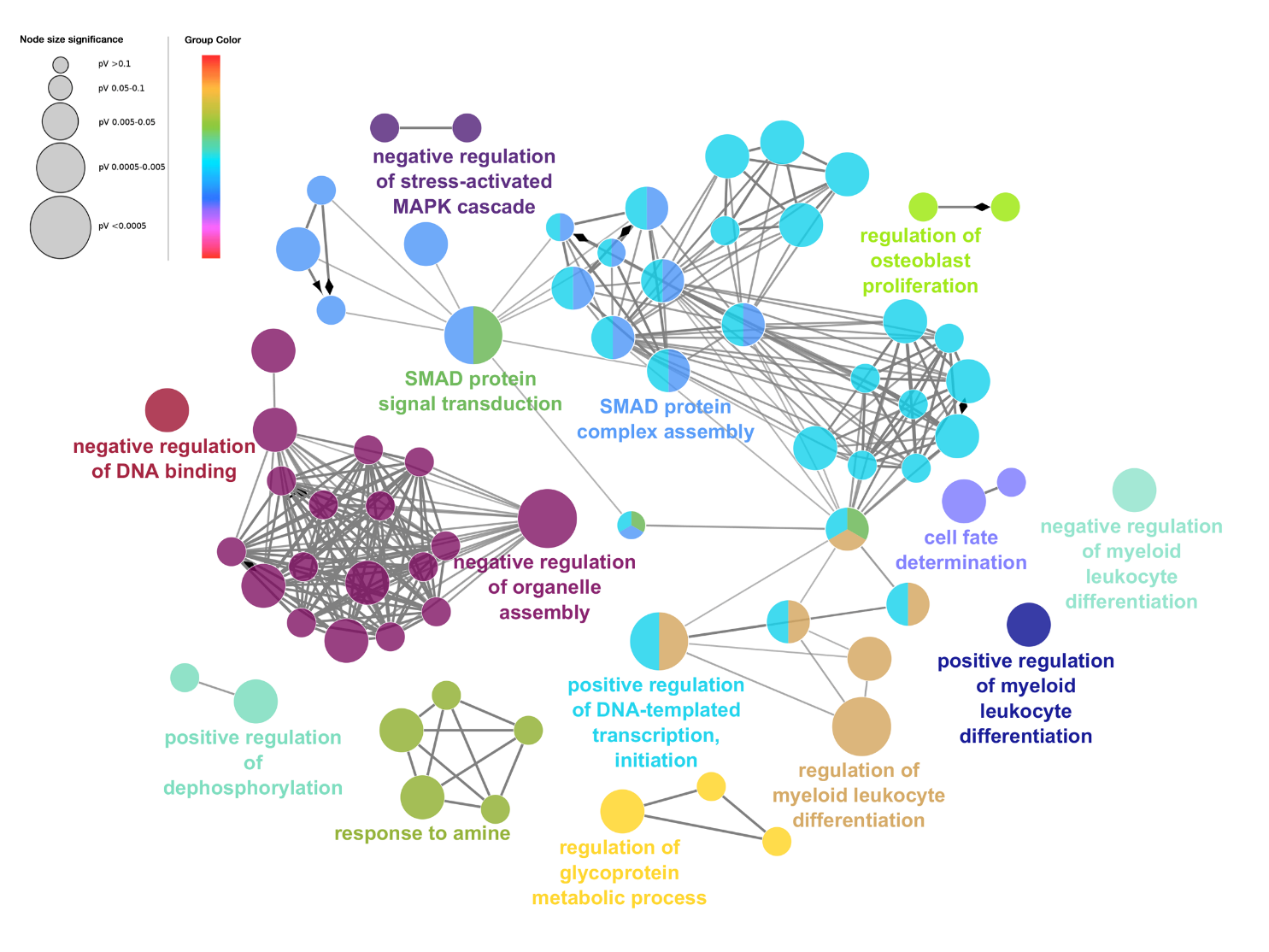

Supplement: Supplementary file 2 — Additional file 2: Figure S1. Biological networks and Enriched gene ontology pathways identified by the functional annotation analysis. Enrichment network of the shared candidate disease genes and their upstream regulator based on biological processes using ClueGO Cytoscape plugin. Hyper-geometric (right-handed) enrichment distribution tests, with a p-value significance level of ≤ 0.05, followed by the Bonferroni adjustment for the terms and leading term groups were selected based on the highest significance. The node size and deeper color indicates greater significance of the enrichment. [file 12967_2018_1504_MOESM2_ESM.docx]
